# Commentary: Why Do You Believe in God? Relationships between Religious Belief, Analytic Thinking, Mentalizing and Moral Concern

**DOI:** 10.3389/fpsyg.2017.00607

**Published:** 2017-04-25

**Authors:** Konrad Talmont-Kaminski, Adrian D. Wojcik

**Affiliations:** ^1^Psychology Faculty, University of Finance and ManagementWarsaw, Poland; ^2^Faculty of Humanities, Nicolaus Copernicus UniversityTorun, Poland

**Keywords:** religiosity, empathizing, socially desirable responding, prosociality, psychopathy

Jack et al. (2016) argue that empathizing and religious beliefs are robustly associated and that this connection cannot be explained by socially desirable responding. Interestingly, however, the data Jack et al. obtained can be re-examined to show that socially desirable responding does mediate the relationship between empathizing and frequency of religious practice. This is a very surprising difference between self-reported religious belief and religious practice which could help to locate Jack et al.'s results within the broader discussion as well as having potential significance for future methodology in the area.

Jack et al. obtain the key result on the basis of study 8 in their paper, which uses a large sample of responses to questionnaire-based measures of empathizing, religious belief, religious practice, and socially desirable responding. Using the Crowne-Marlowe (Crowne and Marlowe, 1960) measure of socially desirable responding, they are able to show that while empathizing and religious belief are correlated, this correlation is not mediated by either of the sub-measures—attribution and denial—that Crowne and Marlowe identified. Interestingly, by re-examining the data Jack et al. provide, it is possible to see that what is true of religious belief does not hold for religious practice. In that case, the connection with empathizing is mediated by different forms of social desirability. The whole mediated model with standardized coefficients is illustrated in Figure 1.

**Figure 1:**
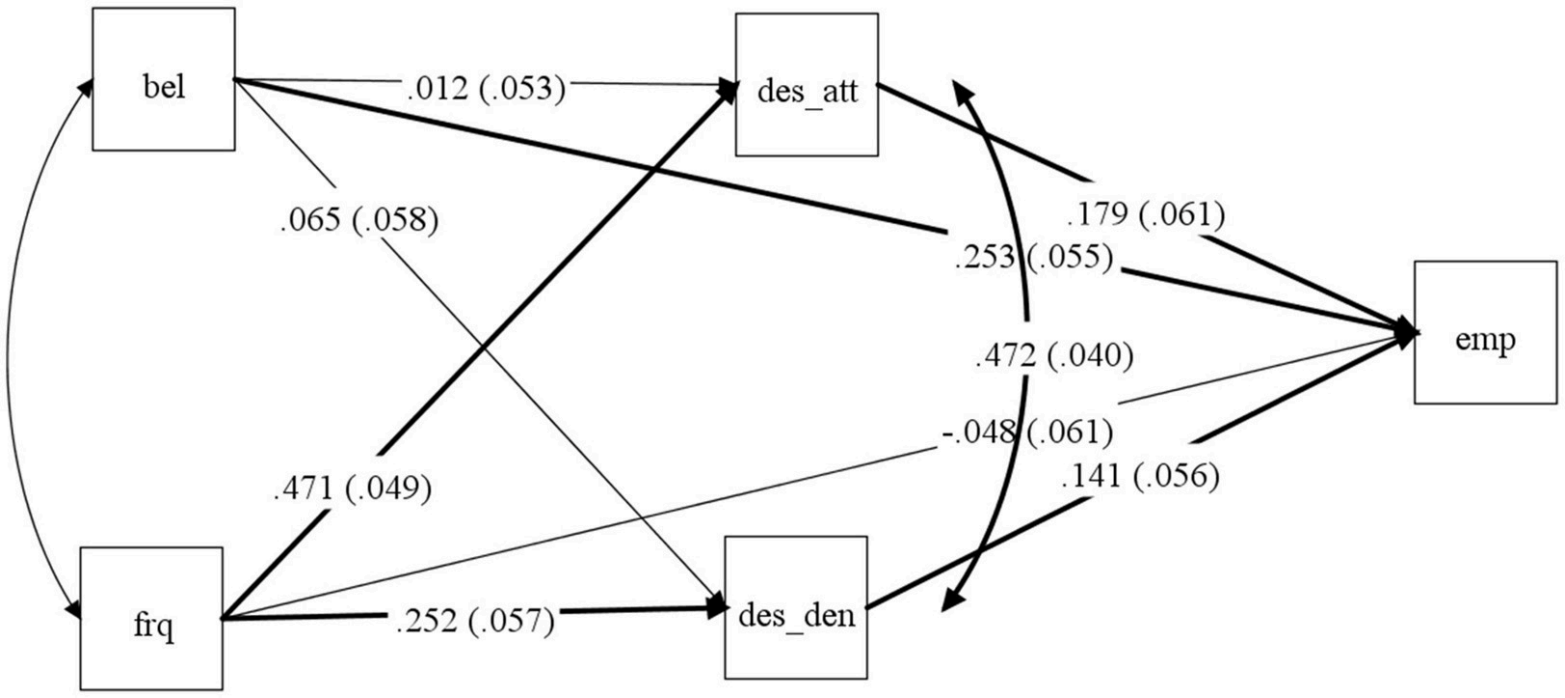
**The full mediation model for the relation between religious beliefs, religious attendance, and empathy**. *Bel*, religious beliefs; *Frq*, frequency of religious practices; *Emp*, empathy; *Des_att*, desirability (attribution measure); *Des_den*, desirability (denial measure). Bolded lines were used to identify significant paths. All path coefficients are standardized.

The model shows that when we control for shared variance of two measures of religiosity—religious beliefs and religious practices—the empathic concern is explained directly only by religious beliefs. The total effect of religious practices is mediated via different forms of social desirability. We examined an indirect effect using bootstrapping analyses with 50,000 re-samples to estimate effect sizes as well as their 95% confidence intervals. Bootstrapping analysis confirmed a significant indirect effect of religious practices on empathic concern through the attribution measure of social desirability (standardized estimate = 0.08 [0.02, 0.15]) and through the denial measure of desirability (standardized estimate = 0.04 [0.01, 0.08]).

While the additional result goes beyond the focus on religious belief that Jack et al. have, it does help to locate their study in the broader discussion of the relationship between religiosity and prosociality. In a recent review of this literature, Shariff (2015) argues that while religiosity is associated with higher self-reported prosociality, it is not associated with behavioral measures of prosociality. The Jack et al. study, showing a connection between self-reported levels of empathizing and religious belief, seems to be in line with this result. However, this is before the significance of socially desirable responding is taken into account.

Shariff thinks that the difference between self-reported and behavioral measures is at least in part due to “a tendency for the religious to be higher in impression management and self-enhancement.” This view also receives support from Sedikides and Gebauer's (2010) review of studies connecting religiosity and socially desirable responding, on the basis of which they conclude that self-reported religiosity is partly due to attempts to self-enhance in the context of societies in which religiosity is viewed positively.

Jack et al.'s result that socially desirable responding does not mediate in the relationship between empathizing and religious belief runs counter to those results. At least in this case, impression management and self-enhancement do not appear to play a role in connecting self-reported measures. So, the fact that the data gathered by Jack et al. shows socially desirable responding playing this role in the case of empathizing and frequency of religious practice is particularly interesting. Given that these are two considerations typically taken into account to measure religiosity and that neither Shariff nor Sedikides and Gebauer distinguish between them in their studies, it might be that Jack et al. have happened upon a methodologically important difference. In particular, if their results were to stand up to further scrutiny, it might be that problems with validity of self-reported religiosity could be lessened by asking about religious belief rather than religious practice.

This interpretation, however, can only be preliminary due to a couple of problems with Jack et al.'s methodology. By using Mechanical Turk to recruit their subjects from any country, Jack et al. could not control whether those subjects live in countries where Sedikides and Gebauer would expect self-reporting religiosity to be an element of self-enhancement (see also Stavrova and Siegers, 2013 for effect of social enforcement on the religiosity-prosociality connection). Also, they could not control the immediate environment the subjects were in while responding, thereby making it possible the subjects were being primed by religious elements in that environment—something that is known to affect prosocial responding.

## Author contributions

KT: co-wrote the commentary. AW: carried out the statistical analysis and co-wrote the commentary.

### Conflict of interest statement

The authors declare that the research was conducted in the absence of any commercial or financial relationships that could be construed as a potential conflict of interest.
